# The Emotional Impact of Educational Productivity Videos on YouTube: A Global, Cross-Sectional Survey

**DOI:** 10.7759/cureus.43989

**Published:** 2023-08-23

**Authors:** Shaun Andersen, Deepal Patel, Andy Nguyen, Prerak Juthani, Kinza Hussain, Joshua Chen, Martin Rutkowski

**Affiliations:** 1 Medical Education, University of Nevada Las Vegas School of Medicine, Las Vegas, USA; 2 Medical Education, Medical College of Georgia at Augusta University, St. Augusta, USA; 3 Internal Medicine, Stanford University Hospital, Palo Alto, USA; 4 Medical Education, University of New England College of Osteopathic Medicine, Biddeford, USA; 5 Medical Education, Sidney Kimmel Medical College at Thomas Jefferson University, Philadelphia, USA; 6 Neurosurgery, Augusta University, Augusta, USA

**Keywords:** motivation, student, youtube, productivity, social media

## Abstract

Introduction

YouTube is the most popular video-sharing website, and many students use it as a resource to find educational content. One type of video category is “productivity,” in which the creator teaches viewers how to lead a more productive lifestyle by sharing ways to maximize studying, reshape daily habits, or set achievable goals. Little research has been conducted on whether these videos actually promote positive or negative feelings among viewers.

Methods

A survey was created through Qualtrics and shared through YouTube and Instagram. The survey asked about exposure to productivity videos and also asked individuals to share their experiences with consuming productivity-related educational content on YouTube. Survey items asked students to rate the helpfulness of these videos and share their feelings about the content. Respondents were asked to share whether YouTube videos on productivity made them feel anxious, motivated, inspired, neutral/indifferent, or inadequate. Participants were also asked to rate how helpful they found productivity videos on YouTube (1-10, with 10 being most helpful). The survey included free response sections to assess viewers’ perceptions and attitudes toward productivity videos.

Results

The cross-sectional survey amassed 595 responses across 60 countries, with 364 responses coming from individuals within the United States. Of the respondents, 397 of the respondents were female, 177 were male, and 21 preferred not to say or identified as non-binary. The average age of participants was 22 years; 79 were in high school, 174 were in college, 223 were in medical school, and the remainder identified as “other” (graduate school, gap year, etc.). Of the 595 completed responses, 494 reported watching videos on YouTube related to improving productivity; when asked how these videos made them feel, 127 participants answered “anxious,” 357 answered “motivated,” 308 answered “inspired,” 95 answered “neutral/indifferent,” and 97 answered “inadequate.” When rating how helpful they found these videos (1-10), an average score of 6.8 was recorded.

Conclusion

Most viewers feel motivated or inspired by productivity videos on YouTube. Based on the free responses provided by survey participants, productivity videos can be made more effective by showing more relatable routines and demonstrating what viewers should do when goals are not met.

## Introduction

Social media platforms allow information to be spread quickly to vast populations of users [1]. In the last decade especially, the adoption of these platforms has fostered higher levels of learning, development, and communication among educators [2]. Of the various social media sites, YouTube is the most popular video-sharing website in the world, and many students use it as a resource to find educational content [3,4]. Retrieving relevant information is dependent on the user’s search terms and the underlying algorithm used to recommend such videos [5].

One type of video category on YouTube is “productivity.” In these videos, the content creator aims to teach the viewer how to lead a more efficient lifestyle by either filming a productive day in their own life or by giving the audience tips to reshape their daily habits and set achievable goals. These habits or goals include, but are not limited to, study strategies, gym routines, weight loss, sleep hygiene, and entrepreneurship. As well-intended as these videos may be, little research has been conducted to evaluate if this type of content actually instills feelings of productivity among the viewers. In fact, there is evidence that these kinds of videos could promote unrealistic lifestyles and negatively affect one’s self-image [6]. Many users use social media for self-esteem and self-growth purposes, and thus, seeing others who are higher-achieving than us can lead to feelings of inadequacy or anxiety [7,8].

Content created by professional associations generally contains trustworthy information; however, not all videos on YouTube are created by experts and official organizations [5]. Many videos are uploaded by “content creators” or “influencers” who have been able to gain the following of hundreds, thousands, or even millions of subscribers [9]. YouTube is an easily accessible platform that allows easier distribution of information with the tradeoff of lack of peer review. There are a variety of methods that have been used to assess YouTube video quality. Some studies use video power index, a set of criteria defined by the Journal of American Medical Association to evaluate the quality of videos containing health-related information [10]. Other studies have used grading criteria based on several video characteristics such as thoroughness, view count, duration on platform, and video length [11]. This discrepancy in evaluating educational content leads to great variation and subjectivity in the quality assessment process, even in content produced by academic physicians [12].

Although many studies have evaluated the quality of educational content on social media, there is limited literature that assesses viewer perceptions and thoughts. The purpose of this study is to evaluate viewer attitudes toward productivity-centered videos on YouTube and use these feelings to create more meaningful digital educational content.

## Materials and methods

Ethics

In this survey-based research study conducted in the United States, Institutional Review Board (IRB) approval was deemed unnecessary due to its classification as a minimal-risk study. A link to an open Qualtrics poll was disseminated through the YouTube and Instagram accounts of four authors of this study. As both platforms used in this study, YouTube and Instagram, belong to the public domain, the survey was accessible to anyone, irrespective of their subscription or account status. None of these accounts used to distribute the survey are private or restricted in access, and there is no collection of replies or comments to these posts gathered to ensure complete anonymity.

Study design

This was a cross-sectional observational study conducted via social media. A survey was designed in Qualtrics in February 2022. The survey aimed to gather information regarding individual experiences with consuming productivity-related educational content on YouTube. All survey respondents were asked about their gender, age, country of residence, and educational level (high school, college, graduate school, or other) to attain baseline demographics. All respondents were first asked if they used YouTube to supplement their education. Answering "No" ended the survey. If "Yes," respondents were asked if they have seen YouTube videos related to improving their productivity. Any respondent who answered "Yes" was then asked to share whether YouTube videos on productivity made them feel anxious, motivated, inspired, neutral/indifferent, or inadequate. Participants were also asked to rate how helpful they found productivity videos on YouTube (1-10, with 10 being most helpful). The survey included free response sections to assess viewers’ perceptions and attitudes toward productivity videos and to provide suggestions for improvement.

On YouTube, the link was shared as a "Community Post," a feature that facilitates direct communication between content creators and their subscribers outside of regular video uploads. This method allowed the survey link to reach the subscribers and non-subscribers of the authors' channels alike. YouTube's policy ensured that the data on who accessed the Community Post or clicked on the survey link remained anonymous, preserving the privacy of the users. Community Posts were taken down 24 hours from the time of posting. Similarly, on Instagram, the survey link was posted as an "Instagram Story," a popular form of temporary content that remains publicly accessible to a user's followers for only 24 hours. As in the case with YouTube, Instagram does not keep track of which users clicked the link to the survey. Using both YouTube and Instagram as platforms to distribute the survey link allowed the authors to capture a more diverse and representative sample of social media users, as different individuals may be active on these platforms at various times.

Statistical analysis

All incomplete responses were excluded from analysis. Data are presented as integers, percentages, and means with standard deviations where appropriate. Total responses, ratings, perceptions, and demographic data were all obtained through Qualtrics survey and are reported with the aid of graphs. Chi-square analysis was used to test for homogeneity of categorical variables (any of “anxious,” “motivated,” “inspired,” “neutral/indifferent,” or “inadequate”).

## Results

**Figure 1:**
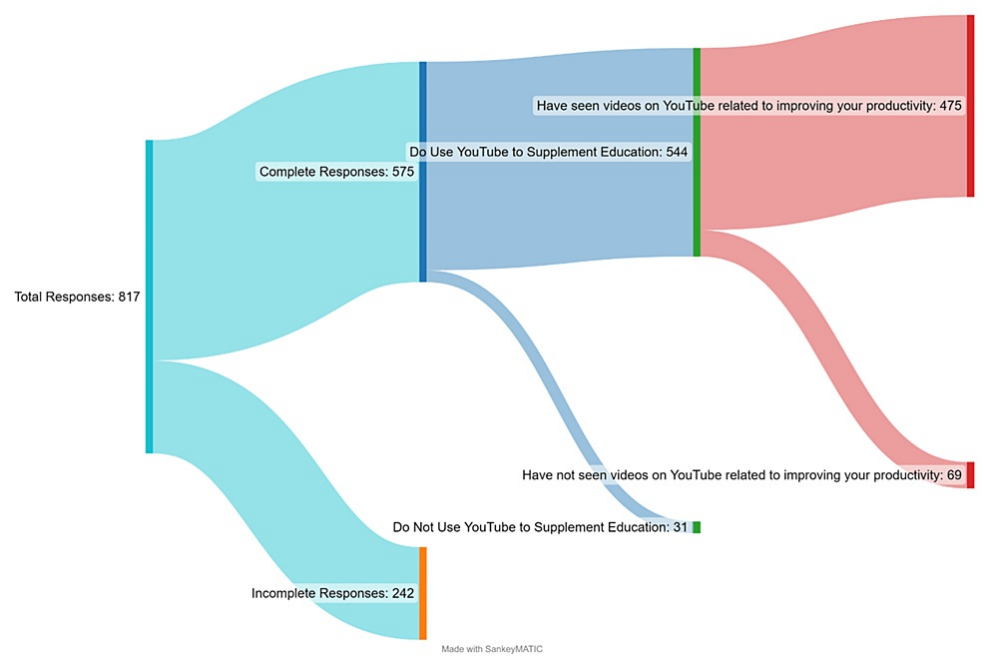
Survey responses

Though we initially received 817 total responses from this survey, 242 were excluded due to incomplete submission or inaccurate answers (i.e., age outside of reasonable boundaries). Furthermore, key aspects of the survey were intended to be sequentially administered (“Do you use YouTube to supplement or enhance yourself or your education?” “Have you seen videos on YouTube related to improving your productivity”). In order to better visualize this flow, we created a Sankey diagram (Figure 1).

As is visualized in this diagram, 575 respondents successfully completed the survey. Of those 575 respondents, 544 (94.6%) reported using YouTube for educational supplement. Of that group, 475 (87.3%) have seen videos related to improving their productivity. Only 69 respondents (12.7%) who use YouTube to supplement their education do not use it to improve productivity.

We also aimed to better characterize the 575 respondents who completed the survey. Using the provided data, a descriptive statistics table was created, as shown in Table 1.

**Table 1:** Descriptive statistics

Descriptive statistics	
Mean age (years) ± standard deviation	22.51 ± 5.46
Sex	
Male	174 (30.3%)
Female	389 (67.6%)
Prefer not to say/non-binary	12 (2.1%)
Total	575
Education level	
High school student	79 (12.9%)
College student	174 (29.9%)
Medical student	223 (37.9%)
Other	113 (19.3%)
Total	575

The mean age of the respondents was 23 years (SD: 5.46). Notably, there was a large female predisposition in response rate (389 females, 174 males, and 12 preferred not to say/non-binary). Education level of respondents skewed toward higher levels of education.

**Figure 2:**
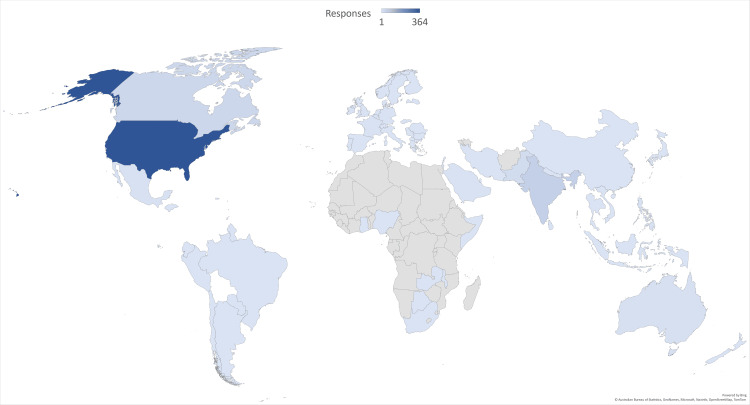
Geographic distribution

We also collected data on geographic distribution of participants to understand the reach of such a survey. Using the data provided, a map chart is shown in Figure 2.

In this figure, darker shades indicate greater response rate, with countries not colored indicating no response. The greatest response rate came from the United States (364), 49 came from India, 31 from Canada, and 18 from Pakistan. All other countries had less than 10 respondents each, and 64 countries were represented in total.

**Figure 3:**
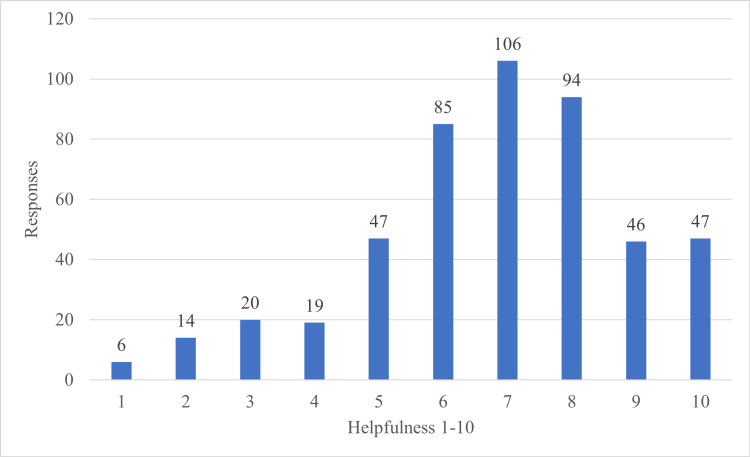
Perceived helpfulness of productivity videos

Assessing the perceived helpfulness of productivity videos was also important to authors. A survey question offered respondents the opportunity to evaluate the helpfulness on a scale of 1-10, with 10 being most helpful. A histogram of responses is shown in Figure 3.

These data indicate that the median helpfulness of improving productivity videos was 7/10 on a 1-10 scale. Overall, these data have a negative skew.

**Figure 4:**
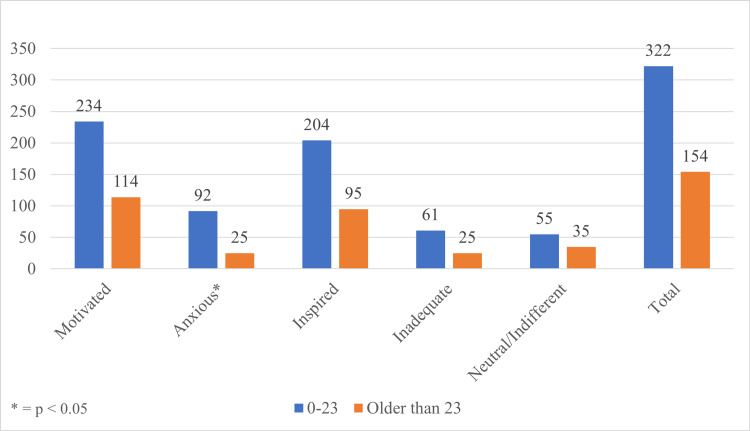
Age-based breakdown of respondent perceptions

Respondents were given the opportunity to answer how they felt after watching productivity-related videos with responses such as “motivated,” “anxious,” “inspired,” “inadequate,” or “neutral/indifferent.” Once all perceptions were totaled, we then divided each group into those younger than or older than the mean age of 23 years. A bar chart of this breakdown is shown in Figure 4.

Our analysis demonstrates that viewers were most likely to feel positive traits such as “motivated” (73.1%) or “inspired” (62.8%) after watching productivity-related videos. Viewers felt negative traits such as “anxious” (24.6%) or “inadequate” (18.1%) at much lower frequencies compared to positive traits. However, younger viewers were significantly more likely than older viewers to feel anxious after watching productivity-related videos (28.6% vs 16.2%, chi-square = 8.554, p = 0.0034). This suggests that while feelings are overwhelmingly positive regarding productivity videos, content creators should be aware that younger viewers, especially those of college-going age and under, may be more susceptible to possible detrimental effects.

Lastly, respondents were given the opportunity to provide their thoughts in free-response format regarding the productivity videos they watch. Two prompts were offered: “Why do you think the videos made you feel this way?” and “What, if anything, would you change about these videos to make them more meaningful?” Representative responses are tabulated in Table 2 and Table 3. In Table 3, responses have been organized into themes. 

**Table 2:** Representative testimonials from free response “Why do you think the videos make you feel this way”

Perception	Quotes
Anxious	They create high expectations which we feel like we can’t reach.
	I feel like I work very little compared to others.
Inadequate	Unrealistic expectations or people being way more productive than I feel I can be. Lots of guilt around not living up to these expectations. Assume that everyone at med school is doing the same thing as the people in the videos and so feeling behind for not being like them.
	It makes me feel like I’m doing something wrong, and my efforts are not near enough, and that will be the reason I failed.
Motivated	It makes me realize that others are going through what I am going through and trying out this method may actually help. It also serves as a foundation for me which I could revise to meet my needs.
	I feel like I’m not alone, and it motivates me to “work alongside them.” That relatability gives motivation. Painting an honest picture with all the struggles shows me that I am not alone. And, of course, I learn that throughout the struggles we have to keep working and we will make it. I think it’s because it’s the reflection of what they are doing, when you see other people doing productive things, it will be a kickstart for you.
Inspired	Looking at people working hard for what they want inspires us that it’s not unachievable, you just need to work hard. I feel capable of reaching my goals when seeing other people doing the things that I want to do. It makes me feel like it is possible to be productive. I find it especially helpful if I am feeling low and unmotivated because [these videos] show a “light at the end of the tunnel.”
	Because they made me feel in control of my success.
Neutral/indifferent	I feel like most videos cover the same topics. Difficult not to compare yourself to others, but gives you insight into improving your own habits.

**Table 3:** Representative testimonials from free response “What, if anything, would you change about these videos to make them more meaningful?”

Theme	Quotes
Transparency	I would maybe give disclaimers like maybe you are generally productive but it’s okay to have moments when you are not productive and are simply living.
Realism/accurate representation of study habits	I think creators being more realistic about the process and journey to becoming a more productive student makes videos more meaningful for me. Seeing students that only show themselves being on top or things and successful makes me compartmentalize them as “perfect” and I am less interested. Seeing students show growth and honest struggles makes them more relatable to me and bridges that gap between creators and the average student.
	It would be helpful for the creator to share some insight on their own personal struggles. If the creator shared some of their own shortcomings or stories of times where things didn’t go as planned, it would make the creator seem more relatable. It would also be encouraging to know that there is room for error and that you don’t have to be perfect to be successful.
	I would have them talk about their failures when trying the techniques they recommend. More realistic perspectives should also be included if possible since med students also get bad grades some days and it takes us time to process those results rather than moving on to the next task at hand.
Video length	I like videos that aren’t 20 minutes long. Keep it short. 8 minutes or less.
	Instead of long videos, replace them with a bullet point blog that is a 3-minute read under the video
Relatability	It would be helpful for the creator to share some insight on their own personal struggles. If the creator shared some of their own shortcomings or stories of times where things didn’t go as planned, it would make the creator seem more relatable. It would also be encouraging to know that there is room for error and that you don’t have to be perfect to be successful. I would add the lazy days. Each of the videos tells us about a person and if we only see the positives, then we imagine that person being nothing but positive. Negative videos on days when the YouTuber is feeling exhausted like everyone else in medicine, if posted, may actually be beneficial to both parties involved. Maybe talk a little bit about failure or those days where everything goes wrong.
Goal achievement	Reminders that your productivity is not tied to your self-worth.

## Discussion

Overall, 83% of completed responses admit to using YouTube to supplement or enhance their education, as well as watch videos related to improving their productivity. Average respondent was predominantly female, from the United States, and 23 years of age. The average helpfulness of productivity videos was rated 6.8/10, and viewers felt mostly motivated and inspired while watching. Suggestions to make such videos more meaningful revolved around themes of transparency and realism, conciseness, brevity, and relatability.

Our findings

Our study shows that respondents feel that productivity videos on YouTube are overall relatively helpful. Most viewers feel like these videos motivate and inspire them to lead more productive and efficient lifestyles. Based on the free responses, much of the reason for this seems to be in line with the effect of social facilitation. Social facilitation is typically seen when participants work on tasks together [13]. However, productivity videos appear to provide this same motivation to viewers as they are able to see another person work through their own tasks. Although feelings toward productivity videos are overall positive, there is still room for improvement primarily with themes revolving around increasing relatability, being more realistic and showing more “down time,” and offering solutions for catching up when goals are not met.

Improving productivity videos

Many of the responses regarding ways to improve productivity videos on YouTube revolved around increasing the relatability of these videos. Since these videos are filmed and edited by the creators, there may be a natural tendency to include the highlights of their days and productivity sessions. Because of this, the breaks between tasks are typically not shown in these videos. This leads to a false belief by the viewers that the content creator is constantly productive without any sort of downtime or struggle between tasks. This can cause the viewers to feel anxious or inadequate and propagate a negative self-image, consistent with concerns for social media use in previous studies [6,14]. Content creators should aim to include more transparency about their breaks and what they are doing when not working on a specific task. Furthermore, since the purpose of productivity videos is to show an audience how to complete their objectives and tasks more efficiently, content creators tend to show themselves accomplishing all of their goals. However, this is not always the case as students may not finish a given project or list of tasks. This leads them to save those goals for the next day along with the other things they need to get done. Although content creators want to serve as an example of what a “highly productive” person looks like, they should include solutions for how to set realistic expectations and accomplishable goals for a given day as well as how to redistribute incomplete tasks on later days. These changes are particularly important when making videos for an audience of college students or younger.

Assessing YouTube video quality

As we have previously discussed, YouTube has been used extensively by educators to facilitate teaching students and even patients [2-4,15]. At this time, assessments of video quality are only conducted after the video has been posted and only if the video has made a large enough impact or meets the search criteria to be included in a study. We propose that a standardized, peer-review process be developed for video creators looking to make educational content. In the same way in which editorial peer-review provides guidance, evaluates methodology and merit, and passes final judgment on the manuscript, a “digital” process ensures that educational videos are assessed prior to public release [16]. This process would be similar to publishing a peer-reviewed manuscript, where experts in the field related to the video topic would review the content, propose revisions, and deem the content as being acceptable for upload or if it should be rejected due to poor content quality. Once a video is accepted, it can be marked with a “peer-reviewed” tag by the website host (i.e., YouTube). This will allow viewers to feel reassured that the media they are consuming has already been fact-checked and peer-reviewed. However, content creators may not want to go through this process to upload their videos. Many creators base their content off of current trends, and awaiting a peer-review process could delay their video publication until a trend is over. On the other hand, educators who post content based on their expertise and do not follow trends may be more motivated to undergo a peer-review process. An elective digital peer-review process maintains creator freedom.

Merits and limitations of social media for survey-based studies

Our study shows the merits of using social media platforms to collect data for survey-based studies. By reaching out to social media users, there is great potential to collect hundreds or even thousands of responses within hours. Many platforms, such as Twitter, have built-in survey capabilities, which have already been used for health-related studies [17]. In our study, we used Qualtrics to generate a link that could be shared on multiple platforms. Furthermore, using a link allowed for multiple users to post the survey rather than just a single account. Doing this allowed us to reach an even wider, broader, and potentially more diverse audience than a single social media platform or user account may have allowed. Although there is potential for reaching a wide audience, the use of social media also comes with its own limitations. For example, the survey link may only be seen by followers or subscribers of a single content creator or small group of content creators (as in this study). This could lead to collecting thoughts from a particular group who are already polarized with opinions that are congruent with the content creator. Respondents of this survey likely already follow the authors because they find their videos helpful, leading to overall positive responses. Furthermore, even if the survey could reach non-following or unsubscribed users via retweets or “recommended” posts via the social media platform’s algorithm, it may only be distributed to users within a particular niche (i.e., medicine, health). With these limitations in mind, the distribution of surveys through social media platforms still serves as an efficient and easy way to collect the general thoughts, feelings, and opinions of viewers in order to make improvements to video content.

## Conclusions

Most viewers feel that productivity videos on YouTube make them feel motivated or inspired to live such lifestyles. However, some viewers report that these videos make them feel anxious or inadequate, with many feeling that these videos are not relatable, lack practicality, are difficult to incorporate into their own lifestyle, or do not include information about what to do when one's own goals are not achieved. Future videos aiming to teach viewers how to be more productive should showcase more relatable schedules and routines, as well as give more insight into what to do when goals are not accomplished.
